# A new page turned, a new beginning

**DOI:** 10.22336/rjo.2023.1

**Published:** 2023

**Authors:** Ruxandra Pîrvulescu

**Affiliations:** * Associate Professor, Ophthalmology Department, “Carol Davila” University of Medicine, Emergency University Hospital, Bucharest, Romania

Every beginning is under the auspices of positive emotions, being a good reason for introspection and for sharing one’s vision for the future. Both journals “Oftalmologia” (from 1994 to 2015) and “Romanian Journal of Ophthalmology” (from 2015) brought an invaluable scientific contribution to ophthalmologists, not only in Romania, but throughout more than 25 countries from all over the world. With small, but confident steps, and with the increased and tireless efforts of the previous Editor-in-Chief and the Editorial Board, Romanian Journal of Ophthalmology turned into an increasingly visible journal, becoming a landmark for Eastern Europe and Romanian doctors and scientists. At present, the journal is indexed by PubMed and has a large scientific contribution especially from foreign authors.

I accepted to take over the position of Editor-in-Chief for Romanian Journal of Ophthalmology from Mihail Zemba, MD, PhD. I am currently an Associate Professor in the Ophthalmology Clinic of the Emergency University Hospital of Bucharest. As an ophthalmologist I had the chance to learn from amazing professors and doctors and to share their values, professionalism, and vision for the future. Having the opportunity of continuing the work of my predecessors, makes me aware of the fact that such a rich and meaningful heritage implies a difficult task and at the same time, a great responsibility towards the readers of the journal and towards the future.

We are presently living a time of continuous change, so we must keep up with it. My colleagues from the editorial team and I are committed to continuing the legacy of our predecessors. Our plans include the following: to increase the quality selection of reviewers and supportive editing of articles, to further improve the quality of the published papers, to keep the journal indexed by PubMed by increasing the contributions of articles from foreign authors and thus, increasing the journal’s visibility and the number of cited articles, to continue and, hopefully, succeed in the efforts of obtaining the journal first impact factor, turning the journal into a pillar of science for the ophthalmologists and scientists throughout Eastern Europe. 

My colleagues from the editorial team and I are embarking in this new journey with the hope of enhancing the journal’s value, visibility, and perspectives and of making it an asset in which medical and scientific worlds can merge, and from which we can all gain as professionals.


**Editor-in-Chief Ruxandra Pîrvulescu, MD, PhD,**
**Associate Professor, Ophthalmology Department,**
**“Carol Davila” University of Medicine,**
**Emergency University Hospital, Bucharest, Romania**

